# ALPPS: PAST, PRESENT AND FUTURE

**DOI:** 10.1590/S0102-67202015000300001

**Published:** 2015

**Authors:** Orlando Jorge M TORRES, Eduardo S M FERNANDES, Paulo HERMAN

**Affiliations:** 1 Universidade Federal do Maranhão (Federal University of Maranhão), São Luís, MA; 2 Hospital Adventista Silvestre, Rio de Janeiro, RJ, Brazil; 3 Universidade de São Paulo (University of São Paulo), São Paulo, SP, Brazil

Complete tumor resection in the liver is the only chance to obtain long-term survival in patients with hepatic tumor or metastasis from other primary cancers. In patients with a large load of tumor within the liver, multiple strategies have been employed to improve resection, especially when a small liver remnant is expected. Staged hepatectomies, in which the surgeon perform partial resection in one side of the liver, and after four to six weeks proceed with the resection of the other side, and strategies to induce hypertrophy of the future liver remnant that include percutaneous portal vein embolization or intraoperative portal vein ligation, have also been largely employed by specialized liver surgery teams.

Hans Schlitt from Regensburg, Germany developed a new procedure, called liver bi-partition, for the first time by chance, in 2007. Planning to perform an extended right hepatectomy in a patient with hilar cholangiocarcinoma - being the future cholestatic liver remnant too small to sustain the patient postoperatively - he decided to perform intraoperatively only a selective hepatico-jejunostomy on the left biliary system, dividing the liver parenchyma along the falciform ligament, thereby completely devascularizing segment 4. Finally, the right portal vein was ligated to induce hypertrophy on segments 2 and 3. On the 8^th^ postoperative day was performed a CT scan and observed a huge hypertrophy of the remnant liver. Recently, de Santibanes and Clavien^2^ proposed the acronym "ALPPS" for Associating Liver Partition and Portal vein Ligation for Staged hepatectomy. The ALPPS procedure has become an advance that represents an important tool to surgically induce fast liver hypertrophy.^1^ ^2^ ^3^

Despite an initial worldwide enthusiasm, the initially reported high mortality rates of 12% by Schnitzbauer et al.­^7^ and 12.8 % by Torres et al.^8^, triggered an intense debate about the safety of this procedure. Several modifications to the originally described ALPPS technique have been reported and careful patient selection became mandatory. The strict selection of patients, not only regarding the cause of the disease but, patients status performance and technical aspects, lead a few groups to perform ALPPS with low or even no mortality.^7^ ^8^

ALPPS became a controversial issue in liver surgery being a subject of debate in many surgical meetings. In last February, the first international consensus meeting on ALPPS, organized by Karl Oldhafer and Thomas Van Gulik, took place in Hamburg, Germany. Liver surgeons from all over the world with experience in ALPPS and many critics of the procedure were present. Five experienced surgeons in ALPPS technique represented Brazil.

In that meeting, several issues were discussed as the indications for ALPPS, technical aspects, hypertrophy, laparoscopy, morbidity, Klatskin tumor, hepatocellular carcinoma, portal vein embolization and two-stage hepatectomy. At the end of the meeting, some recommendations were elaborated.

All patients with indication for ALPPS should be discussed in a multidisciplinary meeting. Inclusion criteria are patients with extensive bilobar colorectal liver metastases, needing an extended hepatectomy, a feasible R0 resection, a predicted future liver remnant <30%, no evidence of extrahepatic disease and complete or partial response to systemic chemotherapy. ALPPS should be mainly indicated for patients that have to undergo a right trisectionectomy. ALPPS is indicated in selected patients with hepatocellular carcinoma and cholangiocarcinoma, but the procedure has higher mortality than for colorectal liver metastases.

Others indications included findings during surgical exploration; so, is necessary to decide intraoperatively in cases with unexpected tumor extension in the future liver remnant or when the future liver remnant volume is adequate but with a macroscopically diseased parenchyma. Failure of portal vein embolization or portal vein ligation, as a rescue surgery has been considered one of the best indication for ALPPS. It is an alternative to conventional two-stage hepatectomy and, comparing with portal vein embolization or portal vein ligation, can lead to a significant and quick growth of the future liver remnant. Age over 60 years, presence of jaundice/cholestasis and additional procedures like pancreaticoduodenectomy are associated with higher morbidity and mortality.^6^

The ALPPS technical aspects group recommended that the middle hepatic artery and middle hepatic vein, if not involved by tumor, should be preserved during the liver partition to avoid ischemic injury and to decrease congestion of the segment^8^. There is no evidence to support coverage of the partition area of the liver in order to decrease adhesions or avoid bile leaks. Ligation of the bile duct in the deportalized liver during the first stage in an attempt to induce hypertrophy of the future liver remnant should be avoided, because ALPPS morbidity is attributed in many cases to biliary leak and the resulting septic complications. Atypical resections of additional metastases (1-3) in the future liver remnant should be performed during the first stage. Use of a loop surrounding hilar structures and hepatic veins during the first surgery, are useful tools for the second stage of the procedure.

An interval o 7-14 days between both procedures in patients with stable condition is also recommended. The short interval to reoperation for ALPPS and the earlier removal of tumor burden improves oncologic outcomes as patients complete the second stage (R0 resection) in more than 90% of the cases (compared to 70% in staged hepatectomies).

Partial partition of the liver during the first stage (named p-ALPPS) has become an interesting option to decrease morbidity after the first stage. This partial partition in the first stage requires more liver transection and liver mobilization during the second stage making it more complex. Partial ALPPS needs to be explored and compared with the classical ALPPS; no recommendations can be given at this moment.

Correlation between volume and function is difficult not only in ALPPS but for all extended liver resections. Functional tests after the first stage seems to be more useful than volumetric studies. Hepatobiliary scintigraphy looks promising due to its capacity to provide simultaneous volumetric and functional information. However, the value of hepatobiliary scintigraphy awaits further clinical assessment in ALPPS.

ALPPS is a novel challenging technique that must pass the test of time. It requires collective experience from specialized centers, refinement of indications and technique in order to reduce the morbidity and mortality rates.
